# Supplementary figures and images for: Weighted Gene Correlation Network Meta-Analysis Reveals Functional Candidate Genes Associated with High- and Sub-Fertile Reproductive Performance in Beef Cattle
Source: Genes (Basel). 2020 May 12;11(5):543. doi: 10.3390/genes11050543 (PMC7290847; doi:10.3390/genes11050543)

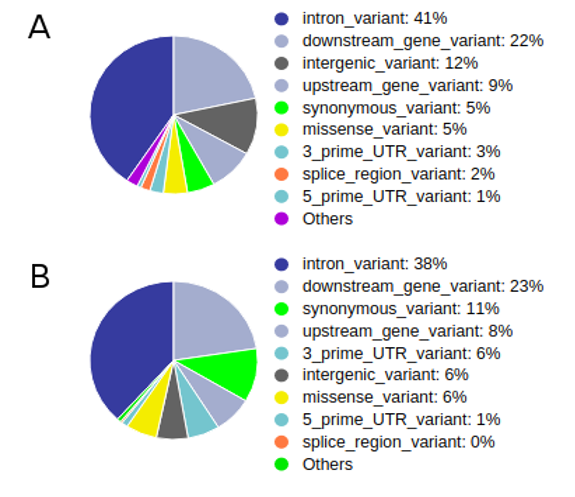

Supplement: Supplementary file 1 [file genes-11-00543-s001.zip › Supplementary_Figure1.png]

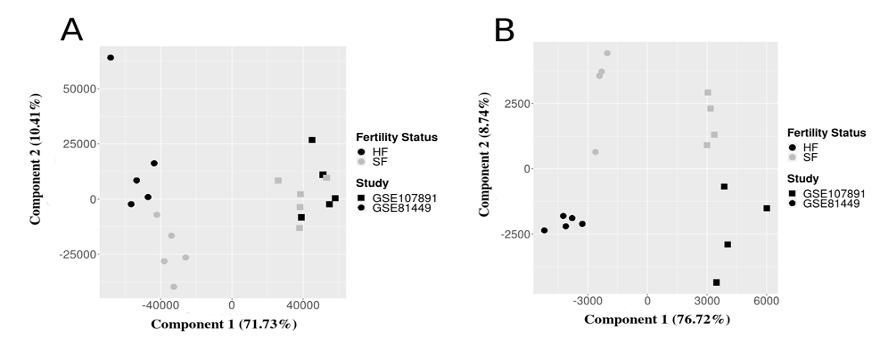

Supplement: Supplementary file 1 [file genes-11-00543-s001.zip › Supplementary_Figure2.png]
